# Melt‐Extrusion‐Based Additive Manufacturing of Transparent Fused Silica Glass

**DOI:** 10.1002/advs.202103180

**Published:** 2021-10-20

**Authors:** Markus Mader, Leonhard Hambitzer, Phillip Schlautmann, Sophie Jenne, Christian Greiner, Florian Hirth, Dorothea Helmer, Frederik Kotz‐Helmer, Bastian E. Rapp

**Affiliations:** ^1^ Laboratory of Process Engineering NeptunLab Department of Microsystems Engineering (IMTEK) Albert Ludwig University of Freiburg Freiburg 79110 Germany; ^2^ Freiburg Materials Research Center (FMF) Albert Ludwig University of Freiburg Freiburg 79104 Germany; ^3^ 3D‐Figo GmbH Lippestraße 20 Salzkotten 33154 Germany; ^4^ Gisela and Erwin Sick Chair of Micro‐optics Department of Microsystems Engineering (IMTEK) Albert Ludwig University of Freiburg Freiburg 79110 Germany; ^5^ Institute for Applied Materials (IAM) Karlsruhe Institute of Technology (KIT) Karlsruhe 76131 Germany; ^6^ Glassomer GmbH Georges‐Köhler‐Allee 103 Freiburg 79110 Germany; ^7^ FIT Freiburg Center of Interactive Materials and Bioinspired Technologies Albert Ludwig University of Freiburg Freiburg 79110 Germany

**Keywords:** 3D printing, additive manufacturing, fused deposition modeling, fused feedstock deposition, fused silica glass, multimaterial printing, nanocomposites

## Abstract

In recent years, additive manufacturing (AM) of glass has attracted great interest in academia and industry, yet it is still mostly limited to liquid nanocomposite‐based approaches for stereolithography, two‐photon polymerization, or direct ink writing. Melt‐extrusion‐based processes, such as fused deposition modeling (FDM), which will allow facile manufacturing of large thin‐walled components or simple multimaterial printing processes, are so far inaccessible for AM of transparent fused silica glass. Here, melt‐extrusion‐based AM of transparent fused silica is introduced by FDM and fused feedstock deposition (FFD) using thermoplastic silica nanocomposites that are converted to transparent glass using debinding and sintering. This will enable printing of previously inaccessible glass structures like high‐aspect‐ratio (>480) vessels with wall thicknesses down to 250 µm, delicate parts including overhanging features using polymer support structures, as well as dual extrusion for multicolored glasses.

## Graphical abstract

Thermoplastic silica nanocomposites allow additive manufacturing of transparent fused silica glass via melt‐extrusion‐based processes like fused deposition modeling and fused feedstock deposition followed by a subsequent sintering process. This enables the manufacturing of transparent high‐aspect‐ratio glass structures with intricate features, complex 3D glass components, as well as multicomponent glasses.

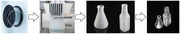

## 1 Introduction

Additive manufacturing (AM) is a rapidly expanding research field being driven mostly by its ability to fabricate complex 3D parts with unrivalled design freedom and fast concept‐to‐prototype cycles. This is why AM quickly established itself as one of the most important methods for prototyping and manufacturing of customized components that are readily applied in various fields ranging from life science applications, products for automotive and aerospace industry, to energy storage devices and architectural designs.^[^ ^1^, ^2^, ^3^, ^4^ ^]^ While AM was mainly introduced for polymers, the processing of ceramics, metals, and even glasses became possible in recent years.^[^ ^4^, ^5^, ^6^, ^7^ ^]^ Glasses and specifically fused silica glass are of high interest because of their high optical transparency, high mechanical stability, as well as high thermal and chemical resistance. While polymers, ceramics, and metals have been processed by AM over three decades, glasses have just recently become accessible to AM. AM of transparent glasses can be categorized in direct and indirect printing methods. In direct approaches, glasses are directly printed in the melt: fused deposition modeling (FDM) of glasses has been shown using melt extrusion of molten glass at high temperatures.^[^ ^8^ ^]^ The glass is molten in a high‐temperature kiln and deposited as strands on a movable print platform, allowing 3D printing of large glass components. This process, however, is limited to low‐melting soda‐lime glasses so far. In addition, it needs a specialized high‐temperature printer with temperatures up to 1165 °C and allows only limited resolutions with typical strand diameters in the range of 4.5 mm.^[^ ^8^ ^]^ Due to the high melting temperature of fused silica glass, melt‐extrusion‐based AM has so far not been possible. Alternatively, fibers have been locally melted using a laser to directly shape fused silica glass, which, however, yields parts with limited dimensional accuracy and low resolution.^[^ ^9^ ^]^

Indirect approaches have enabled AM of fused silica glass by printing glass precursors, which are subsequently converted to transparent fused silica glass via debinding and sintering. Using a photocurable nanocomposite resin we have previously shown that fused silica can be shaped via stereolithography (SL) or two‐photon polymerization (2PP) of silica nanocomposites.^[^ ^7^, ^10^, ^11^ ^]^ 2PP and SL enable 3D printing of micro‐ as well as macroscopic transparent fused silica components. However, SL, in general, struggles with printing of large high‐aspect‐ratio structures since residual stresses from polymerization shrinkage during printing and postcuring can result in cracks, delamination, and distortions. These can be reduced by improving the resin formulation and optimizing the printing parameter, but they usually cannot be fully avoided.^[^ ^12^, ^13^ ^]^ A further problem is the need to rinse the printed parts afterward, which may collapse or rupture delicate high‐aspect‐ratio structures.^[^ ^14^ ^]^ In addition, SL does not inherently support multimaterial printing but needs special resin switching procedures and cleaning of the prints when changing materials, making multimaterial components as well as complex geometries with the need for easily removable support structures difficult to achieve.^[^ ^15^ ^]^ In a similar indirect approach, 3D printing of glass has been shown using solution‐based composites for robocasting (RC) and direct ink writing (DIW) that can be converted to glass by subsequent debinding and sintering. While supporting multimaterial printing for production of, e.g., gradient index lenses, these methods are not suited for large high‐aspect‐ratio structures due to viscous flow of the liquid composite materials.^[^ ^16^, ^17^, ^18^ ^]^

Melt‐extrusion‐based AM, with its most prominent representative FDM (also known as fused filament fabrication (FFF)), offers many advantages over the already existing AM methods for transparent fused silica. In this AM process, a thermoplastic polymer is introduced into a printer either as granules or as filaments, molten in a heated print head, extruded through small‐diametered nozzles, and deposited on a print bed in three dimensions, generating 3D objects in a layer‐by‐layer‐based fashion. With typical nozzle sizes of <0.4 mm and layer heights in the range of 100–300 µm, FDM can be used to achieve reasonably high printing resolutions. Due to rapid solidification of the extruded material, large high‐aspect‐ratio structures can be easily fabricated especially if nonplanar slicing is used.^[^ ^19^ ^]^ Furthermore, FDM is one of the most common printing processes on the market, since the printers are easy to use, cost effective, and fast.^[^ ^20^ ^]^ Besides its user friendliness, FDM offers the possibility of multimaterial printing, e.g., for simultaneous printing of different colored materials or the use of a different material for support structures that can be removed easily without the need for manual removal. FDM further enables the so‐called print–pause–print principle, allowing integration of external objects into the final 3D‐printed part.^[^ ^21^, ^22^ ^]^ Fused feedstock deposition (FFD) is a similar method, where thermoplastic granules are printed directly using a screw‐based system and, therefore, circumventing the need for filament fabrication. This makes FFD significantly easier to apply on a larger scale as many interesting feedstock materials are predominantly available as granules.^[^ ^23^, ^24^ ^]^

In this work, we present an indirect melt‐extrusion‐based process for AM of transparent fused silica glass. We show FDM and FFD 3D printing of thermoplastic fused silica nanocomposites, which we recently developed for injection molding, using commercially available melt‐extrusion‐based printers. The printed parts are subsequently converted to transparent fused silica using debinding and sintering at a maximum temperature of 1320 °C. We show various printed fused silica structures highlighting the simplicity and versatility of melt‐extrusion‐based 3D printing. We printed large high‐aspect‐ratio thin‐walled components for applications ranging from chemically stable and lightweight laboratory or pharmaceutical glass containers, customized glass structures for everyday usage to delicate hollow glass components, e.g., for novel lighting concepts. We also show printing of 3D bulk components such as fully functional microfluidic chips facilitating microfluidic prototyping by circumventing complex bonding procedures. In addition, we show multimaterial printing with doped nanocomposites yielding multicolored glasses, multimaterial printing with polylactic acid (PLA) support structures for printing of otherwise inaccessible overhanging structures as well as integration of external components using print–pause–print. This novel process represents a major addition to the field of glass 3D printing bypassing many of the disadvantages associated with the already known methods for AM of glasses with possible applications ranging from scientific research, rapid prototyping to components for everyday usage.

## 2 FDM Printing of Thermoplastic Silica Nanocomposites

FDM printing is one of the most commonly used 3D printing methods due to the simplicity and versatility of the process itself. However, despite its wide use, the development of novel filament materials is challenging even for pure polymers since there is a delicate balance between low melt viscosity as well as high mechanical stiffness and high flexibility of the polymer filaments to be met.^[^ ^25^ ^]^ Composite materials, such as metal‐ or ceramic‐filled materials, are even more difficult since the composite filaments are often inherently brittle and show a high melt viscosity.^[^ ^4^ ^]^ Therefore, to successfully print silica nanocomposites using FDM, the composition of the fused silica nanocomposite has to allow preparation of a filament that is flexible enough to circumvent breakage but still mechanically sufficiently stiff to generate high pressure on the molten feedstock without filament buckling. In addition, the material must have a low viscosity to prevent nozzle clogging.

**Figure 1:**
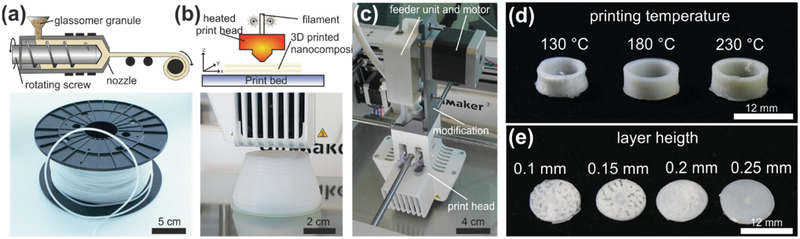
FDM printing of thermoplastic silica nanocomposites. a) Schematic of the filament production process and a spool of the produced silica nanocomposite filament. b) Schematic of the FDM printing process and a picture of printing a vase using the filament shown in panel (a). c) Modification of the FDM printer. The feeder unit and motor were fixed directly on top of the print head to improve filament handling during printing. d) Printing of ring‐shaped components with different printing temperature to determine the best print head temperature setting. Printing at 180 °C yielded the best results with high shape accuracy. Printing at lower temperatures (130 °C) showed a deformation of the ring due to low material extrusion. Printing at high temperatures (230 °C) showed yellow coloring of the printed feedstock, indicating thermal degradation of the binder. e) Printing of flat, spherical plates with different layer heights (0.1–0.25 mm) to determine best layer height settings for defect‐free printing results and highest achievable printing resolution. Layer heights smaller than 0.2 mm show significant defects due to low material flow, in form of holes and gaps that prohibit printing of dense and defect‐free components. Layer heights bigger than 0.25 mm show homogeneous printing results and allowed printing of a dense and defect‐free plate.

For the 3D printing of fused silica, we used a Glassomer nanocomposite, originally developed for injection molding, consisting of polyethylene glycol (PEG), polyvinyl butyral (PVB), and silica nanoparticles with solid loadings up to 60 vol%.^[^ ^26^ ^]^ For the preparation of the nanocomposite, silica nanoparticles with a mean diameter of 100 nm were mixed into a solution of PEG, having a molar mass of 4000 g mol^−1^, and PVB in water using a laboratory dissolver. After drying the nanocomposite at 65 °C to remove the solvent, the nanocomposite was plasticized and extruded using a twin‐screw compounder at 130 °C to obtain a continuous filament. To address the issue of mechanical stability, we choose a filament diameter of 2.85 mm, yielding a filament with high strength and high flexibility. To decrease the viscosity of the Glassomer feedstock, we used nanocomposites with a reduced fused silica solid loading of 40 vol% for preparation of FDM printable filaments. In addition to a reduction of the viscosity, a decrease in solid loading additionally yielded a more flexible and less brittle filament. **Figure** **1** shows the process of FDM 3D printing of the fused silica nanocomposite. To prepare a printable filament, the thermoplastic nanocomposite was extruded at a temperature of 130 °C through a 2.85 mm nozzle with speeds up to 1.5 kg h^−1^. Using this setup, a continuous flexible filament, with a uniform diameter of 2.76 ± 0.03 mm (ten measurements over a length of 10 m), could be produced. The flexible filament could be continuously spooled and unspooled on commercial filament rolls without breakage even at room temperature (see Figure 1a,b).

To print the thermoplastic nanocomposite filament, we used a commercial FDM printer equipped with a 0.4 mm nozzle. For improved filament handling, we modified the FDM printer by removing the feeding motor unit from its original place at the back of the printer (bowden type) and placing it directly on top of the print head (direct drive), as shown in Figure 1c. This shortens the path length that the filament has to take from the feeding motor to the print head and ensures that the feeding motor applies its pressure perpendicular to the print head, therefore reducing the risk of filament breakage.^[^ ^27^ ^]^ Using this setup, filaments made from Glassomer granules with a solid loading of 50 vol% led to clogging of the printing nozzle due to the high melt viscosity.^[^ ^26^ ^]^ Glassomer granules with a silica solid loading of 40 vol% yielded successful and reproducible printing results. To accommodate for the increased heat capacity induced by the high silica solid loading, the printing temperature was gradually increased to find the best print settings, starting from the standard temperature for extrusion at 130 °C. To evaluate the print quality in dependence of the printing temperature, we printed ring‐shaped structures using different print head temperatures and analyzed the quality of the printed parts (see Figure 1d). At low temperatures of about 130 °C, the material showed bad shape accuracy. Slight bulging of the ring wall was observed, which can be explained by a low material flow rate due to high viscosity resulting from a too low melt temperature. After increasing the temperature of the print head up to 180 °C, good material flow was observed, and reproducible 3D printing results with sharp and perpendicular walls were obtained. Temperatures higher than 180 °C also yielded good printing results, but the printed parts show slight yellow coloring, indicating a degradation of the polymer binder matrix (see Figure 1d). The print bed temperature was set to 60 °C giving a good adhesion of the component to the print bed. The cooling fan located in the print head was set to 100% to cool down and solidify the printed material as quickly as possible allowing us to 3D print complex‐shaped components without warping. A high printing speed of up to 60 mm s^−1^ was found to be beneficial for reproducible printing results since the feedstock shows shear thinning behavior, and a higher printing speed therefore reduces the viscosity of the nanocomposite.^[^ ^26^ ^]^ To improve the printing resolution, we analyzed the influence of the layer height printer setting on the printing procedure. For this we printed disk‐like structures with different layer heights and analyzed the visual appearance of the printed disks in regard of printing quality and defects (see Figure 1e). The minimum layer height for successful FDM printing was found to be 200 µm resulting in a uniform, defect‐free disk. Smaller layer heights resulted in structural defects (holes and gaps), which can be explained by an inhomogeneous material flow and clogging of the nozzle due to the combination of high viscosity and lower material extrusion rates. For a higher reproducibility of printing quality, we choose 0.25 mm layer heights for all further prints using the 0.4 mm nozzle.

We characterized the mechanical properties of the FDM‐printed green parts with different strand orientations by tensile testing to determine the influence of strand orientation on the strand‐on‐strand adhesion strength. Injection‐molded nanocomposite specimens were measured as reference. All specimens showed a brittle fracture. Exemplary stress–strain curves are shown in Figure S1 (Supporting Information) and the results of the analysis are summed up in Tables S1–S3 (Supporting Information). It was found that the mechanical properties of the FDM‐printed nanocomposite are mostly independent of the strand orientation (parallel or perpendicular to the strain direction). In nanocomposites with strands printed parallel to the strain direction, the mechanical stability of the nanocomposite itself is the dominant factor while in specimens printed with strands perpendicular to the strain direction the strand‐on‐strand adhesion is the more dominant factor determining the mechanical stability. Since both orientations gave similar results for the tensile modules (≈110–130 MPa), the elongation at breakage (≈3.2%), and breaking stress (≈5.3–5.6 MPa), it can be concluded that the strength of the strand‐on‐strand adhesion is similar to the mechanical strength of the nanocomposite itself. Injection‐molded specimens showed a slightly higher tensile modulus (≈316 MPa), elongation at break (≈3.5%), and breaking stress (≈12 MPa) than FDM‐printed specimens, which can be explained by the fact that the FDM inherent‐strand‐based construction of the specimens yields high notch stresses weakening the mechanical stability overall.

The printed green parts were then debinded using a two‐step protocol. For this, the green parts were first immersed in water to dissolve the PEG component. Afterward, the remaining binder was thermally decomposed in a second debinding step. Due to the first aqueous debinding step, high heating rates of 1 K min^−1^ and short holding phases of 1 h at 270, 460, and 600 °C, respectively, could be employed yielding defect‐free brown parts that were subsequently sintered in vacuum at 1320 °C to dense and transparent fused silica glass. Alternatively, the brown parts can be converted to transparent fused silica glass by sintering under atmospheric pressure in air. Fused silica glass obtained by atmospheric pressure sintering has the same optical appearance as vacuum sintered glass (see Figure S2a in the Supporting Information), although there are usually some minor changes in its optical properties, especially in the UV region, as we have shown before.^[^ ^28^ ^]^ However, since the inherent layer structure of FDM‐printed glass decreases the transmission overall compared to optical quality glass prepared by soft replication, this influence is not noticeable anymore, as we could show by comparing ultraviolet–visible (UV–vis) spectra of glass samples sintered in vacuum and under atmospheric pressure (see Figure S2b in the Supporting Information).

### 2.1 FDM Printing of Single‐Wall Fused Silica Structures

**Figure 2:**
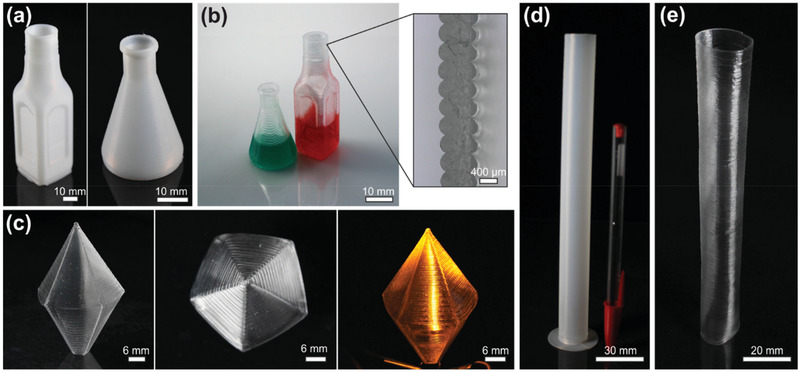
FDM printing of high‐aspect‐ratio single‐wall structures in fused silica glass. a) FDM‐printed green parts of exemplary high and thin‐walled single‐wall bottles printed with the 0.8 mm nozzle and 0.5 mm layer thicknesses. The structures shown were printed in less than 10 min. b) The FDM‐printed single‐wall components were subsequently converted to transparent fused silica glass and filled with dyed water to show leak proofness. The inset shows a magnification of the cross section of the FDM‐printed single‐wall fused silica glass that was printed with a 0.8 mm nozzle. No defects between the separate strands can be observed showing a good strand‐on‐strand adhesion. c) An FDM‐printed and FDM‐sintered hollow single‐wall bipyramidal fused silica glass component shown from different angles to demonstrate high shape accuracy even for components with high slopes. The hollow fused silica glass component can be illuminated with an light‐emitting diode (LED) within for lighting applications. d) Green part of a single‐wall tube with the highest achievable aspect ratio of 600, limited only by the printer's size. A regular pen was included as a size reference. e) Sintered single‐wall tube in fused silica with the highest achievable aspect ratio of 480 and a wall thickness of 250 µm. The picture was taken at a slightly oblique angle to additionally show the thin walls of the sintered high‐aspect‐ratio fused silica glass tube.

One advantage of FDM printing is the fact that extruded material solidifies rapidly upon deposition and cooling, allowing facile printing of high‐aspect‐ratio structures without deformation by viscous flow. To show this, we printed several structures (bottles, tubes, and hollow bipyramids) using the spiralize function of the FDM printer, which prints the outer contour of an object in a continuous manner by extruding only a single strand that spiralizes upward gradually (**Figure** **2**). Using the 0.4 mm nozzle, we could demonstrate printing of up to 20 cm high single‐wall structures with wall thicknesses of about 330 µm in the green part and 250 µm in the sintered glass part, respectively. Larger nozzle sizes (0.6 and 0.8 mm) can be used to increase the wall thickness of the single‐wall components allowing wall thicknesses of 0.6 and 0.8 mm (corresponding to 0.44 and 0.59 mm after sintering) without changing the actual printing procedure. For FDM printing with the larger nozzle sizes, the layer thickness was increased up to 0.4 and 0.5 mm, respectively, allowing for an additional significant decrease in printing duration. The exemplary bottles shown in Figure 2a were printed in less than 10 min using a 0.8 mm nozzle with layer heights of 0.5 mm.

Upon deposition, the nanocomposite strands bond to each other without any cracks or delamination. To show this, the FDM‐printed and sintered glass bottles were filled with colored water, and no leakage could be observed (Figure 2b). In addition, we show a microscope image of a single‐wall cross section demonstrating good strand‐on‐strand adhesion without any delamination (Figure 2b). Fused silica structures like this might be used for various applications including customized bottles, vases, or lighting applications for everyday usage as well as artistic objects or high‐performance customized labware (Figure 2b,c). The maximum printing size was only limited by the available printing space, as we have demonstrated by printing a single‐wall tube with a height of 20 cm and a wall thickness of 330 µm resulting in an aspect ratio of about 600 (Figure 2d). Sintering of the printed high‐aspect‐ratio structure was only limited by the available space in the furnaces. We show a sintered transparent fused silica glass tube with 250 µm wall thickness and a final height of 12 cm resulting in an aspect ratio of about 480 (Figure 2e).

### 2.2 FDM Printing of Bulk Fused Silica Glass Components

**Figure 3:**
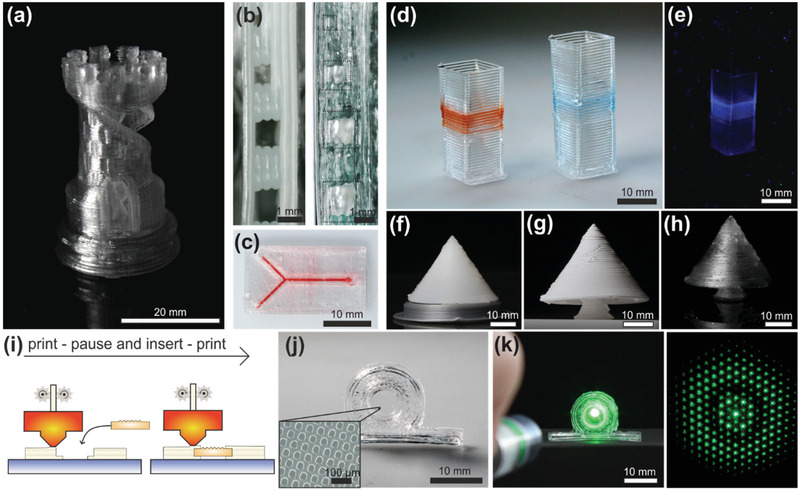
Scope of melt‐extrusion‐based AM of transparent fused silica glass. a) An FDM‐printed 3D tower in fused silica glass. The tower was printed with hollow walls (0% infill) to increase the optical clarity of the sintered glass. b) Cross‐sectional view of FDM‐printed rectangular embedded microfluidic channels. The left image shows the green part and the right image shows the same component after sintering to fused silica glass. The sintered part shows channel widths of about 730, 590, 440, and 240 µm demonstrating that microchannels with a minimum resolution of 240 µm can be printed with good dimensional accuracy. c) An FDM‐printed microfluidic chip with an embedded 590 µm wide channel. The channel was filled with dyed water to demonstrate functionality. d) Multimaterial printing using metal‐salt‐doped silica nanocomposite allows fabrication of multicomponent fused silica glass objects having area‐specific properties. The HAuCl_4_‐doped nanocomposites yields a red‐colored glass and doping with Co^2+^ yields a blue coloration. Scale bar: 10 mm. e) A Ce(NO_3_)_3_‐doped filament was introduced into a printed tube. The Ce^3+^‐doped glass results in a transparent fused silica glass that shows luminescence if illuminated with UV light at 254 nm. f–h) FDM multimaterial printing of f) overhanging silica nanocomposite structures using PLA supports, g) which can be removed during thermal debinding yielding a support structure‐free brown part h) that can be subsequently converted to h) fused silica glass. i) Schematic showing the print–pause–print principle to integrate external objects into FDM‐printed components. j) A hot embossed high‐resolution micro‐optical lens array (MLA) customized with an FDM‐printed bracket using the print–pause–print principle and subsequent sintering to fused silica. The inset shows a magnification of hot embossed MLA in fused silica glass. k) The bracket allows the MLA to be placed in an upright position, and illuminating it with a laser (532 nm) shows the characteristic refraction pattern of the MLA.

FDM printing is not limited to high single‐wall structures only. More complex bulky parts with 3D features can be FDM‐printed and FDM‐sintered to fused silica glass as shown in **Figure** **3**a for an exemplary tower having a free standing double helix structure inside. The tower was printed using a 0.8 mm nozzle and a layer height of 0.4 mm. The infill, which is the percentage of volume coverage within enclosed sections of the model, was set to either 100% for full density or 0% for hollow components. An infill somewhere between 0% and 100% as commonly used in FDM printing was avoided due to the fact that the infill structure would be visible in the final transparent glass component. While bulky parts can theoretically be printed with 100% infill and successfully converted to defect‐free fused silica glass afterward, it has to be noted that the transparency of the sintered glass is decreased due to the process‐inherent generation of air inclusion between multiple strands. To improve optical transparency, we printed the tower as a hollow object with 0% infill and a wall thickness of two strands (1.6 mm) yielding a transparent fused silica glass tower with complex 3D features. We further show the possibility of 3D printing fused silica microfluidic chips, enabling a quick and simple way for fabrication of customized microfluidics, e.g., for microanalytical systems or droplet generators.^[^ ^29^ ^]^ A big advantage of AM is the possibility of manufacturing embedded, fully functional microfluidic chips in one step, without the need for complicated bonding procedures.^[^ ^30^ ^]^ To FDM‐print leak proof microfluidic channels, the general printing parameters for the 0.4 mm nozzle with 100% infill were used with small adjustments. The cooling fan was turned off to improve bonding between strands and fabricate a tightly sealed channel. In addition, the first layer closing the channel structure was printed using bridge settings to reduce sagging. For bridges, the material flow rate was decreased by 50%, printing speed was reduced from 25 to 12.5 mm s^−1^, and the cooling fan was set to 100% to accelerate solidifying of the extruded material and therefore inhibits sagging. We printed several straight embedded test channels with a rectangular cross section with different channel widths of 1000, 800, 600, 400, and 200 µm to determine the highest printing accuracy for embedded microfluidic channels. Figure 3b shows the cross sections of the FDM‐printed test channels before and after sintering to fused silica glass, showing well‐defined channel geometries and dimension down to 600 µm channels (corresponding to 440 µm after sintering). Smaller channels could also be successfully printed with good shape accuracy; however, a slight deviation of the actual channel dimension from the original design was observed. The smallest FDM printable channel with a designed channel width of 400 µm showed an actual channel width of about 330 µm (corresponding to about 240 µm after sintering) due to printing inaccuracy resulting from the employed slicing settings 0.27 mm layer heights and 0.35 mm strand width. Figure 3c shows an exemplary FDM‐printed and FDM‐sintered fused silica microfluidic chip with 590 µm channel width, which was filled with colored water, showing a fully functional and leak proof microchannel.

### 2.3 Multimaterial FDM Printing

A major advantage of FDM over other AM methods is that the printing process supports multimaterial printing. During printing, the filament can be changed, either automatically within the print head or by using a printer with several print heads, allowing simultaneous 3D printing of different filaments. In this work, we showed that multimaterial FDM can be used to print two different doped or nondoped glass nanocomposites simultaneously for the fabrication of multicolored glass components. The employed printer uses a dual extrusion print head that is equipped with two separate print heads, which are selected automatically during printing. This allows us to reversibly change the filament type during printing. By using a HAuCl_4_‐doped filament, we could print transparent fused silica components showing red colored features due to the formation of gold nanoparticles during the heat treatment (Figure 3d).^[^ ^31^ ^]^ Printing with CoSO_4_‐doped filaments allowed printing of components with blue colored features (Figure 3d).^[^ ^31^ ^]^ Multimaterial printing with Ce(NO_3_)_3_‐doped filaments yields a colorless fused silica glass including the doped silica glass features that show luminescence behavior if illuminated under UV light (Figure 3e).^[^ ^32^ ^]^

We further showed that multimaterial printing can be used for the fabrication of support structures for components with overhanging features. Therefore, commercial PLA was used as the material for the second filament. The printed PLA support structures could be removed after printing either by solvent‐based removal during aqueous debinding or by thermal decomposition during the thermal debinding step (Figure 3f,g). Both ways allow the removal of the support structures without the need of manual removal which has been shown to be problematic introducing mechanical stresses on the 3D‐printed part that potentially lead to component failure.^[^ ^33^ ^]^ The debinded and support structure‐free part could then be sintered to fused silica glass without any deformation of the overhanging features (Figure 3h).

### 2.4 Integration of External Object in FDM‐Printed Components

A further benefit of melt‐extrusion‐based 3D printing is the possibility of integrating components during the print using the so‐called print–pause–print principle. Here, an object is inserted at the respective position after the print is paused at the right moment (Figure 3i). Afterward, the print is resumed to fully include the external object. This allows a potential combination of high‐throughput manufacturing methods like injection molding or hot embossing and customizable 3D printing as well as the integration of high‐resolution structures that would otherwise not be accessible by FDM printing. We show an exemplary micro‐optical lens array (MLA), fabricated by hot embossing of thermoplastic Glassomer nanocomposite^[^ ^34^ ^]^ that was customized with an FDM‐printed bracket using the print–pause–print principle (Figure 3j,k). The bracket was printed without further changes to the general printing parameters for 0.4 mm nozzles. The print was automatically paused, and the high‐resolution MLA inserted at the moment the contour of the respective cavity in the bracket was finished printing. Afterward the print was resumed to fully include the MLA into the FDM print effectively fusing both parts together. The combined part could then be debinded and sintered to a single transparent fused silica glass component.

## 3 FFD Printing of Thermoplastic Silica Nanocomposites

**Figure 4:**
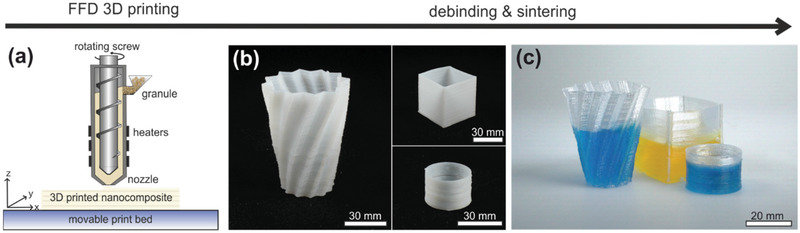
FFD printing of thermoplastic silica nanocomposites. a) Schematic of the FFD printing process. Granules are printed directly by plasticizing in an extruder and 3D deposition of the molten feedstock on a heated print bed, therefore circumventing the need for filament preparation. Using a commercial FFD‐type printer Glassomer feedstock granules with a solid loading of 50 vol% could be 3D‐printed. b) FFD‐printed green parts of complex thin‐walled components with wall thickness down to 800 µm. c) The FFD‐printed components can be sintered to transparent fused silica glass.

While FDM printers are cost effective and easy to use, the process itself is limited in the choice of the applicable materials since processable filaments can be challenging to fabricate. In the case of thermoplastic fused silica nanocomposites, this limits the silica solid loading of the feedstock. A high solid loading, however, would be beneficial for the fabrication of large glass components, reducing shrinkage, and required binder as well as allowing processing more components simultaneously in the same furnace. To increase the solid loading of the printed nanocomposite and to further simplify this 3D printing process, we additionally evaluated FFD of thermoplastic silica nanocomposite (**Figure** **4**). FFD uses an extruder that is mounted on the printer enabling us to melt and extrude granulated materials directly circumventing the need for filament preparation (Figure 4a). The FFD process lowers the mechanical requirements of the materials substantially and allows the printing of feedstock with higher viscosities. We could show FFD printing of thermoplastic Glassomer granules with a solid loading of up to 50 vol%, which was not accessible by FDM printing due to high melt viscosity and low mechanical stability of the filaments. Using a nozzle with a diameter of 0.5 mm and temperatures of around 100 °C, we showed 3D printing of thin‐walled vases with wall thicknesses of down to 800 µm printed at printing speeds up to 70 mm s^−1^ (Figure 4b). The printed parts were subsequently converted to transparent fused silica by debinding and sintering in the same manner as described for the components printed by FDM (Figure 4c).

## 4 Characterization of 3D‐Printed Fused Silica Glass

The properties of fused silica glass prepared by sintering of Glassomer nanocomposites are indistinguishable from commercial fused silica, as has been shown before for the Glassomer process using IM, soft replication, and stereolithography.^[^ ^7^, ^10^, ^26^, ^28^ ^]^ However, for melt‐extrusion‐based printing, there are some exceptions, especially in density, mechanical stability, and transparency of the sintered glass, due to air inclusions generated by the strand‐based construction of the 3D objects. We have characterized and compared the mechanical properties of FDM‐printed and injection‐molded fused silica glass by measuring the bending strength via three‐point bending (see Figure S3 and Tables S4 and S5 in the Supporting Information) as well as the Vickers hardness (see Table S6 in the Supporting Information). FDM‐printed glass shows a bending strength of 67 ± 11 MPa, which is only slightly lower than the bending strength of injection‐molded fused silica glass (90 ± 18 MPa) or commercial fused silica glass (93.7 ± 35 MPa^[^ ^28^ ^]^) showing that although there are some FDM inherent defects present, the glasses are still mechanically strong. Vickers hardness on the other hand was found to be not influenced by the strand‐based construction of FDM‐printed parts giving similar results for FDM‐printed fused silica glass (780 ± 190 HV), injection‐molded fused silica glass (760 ± 160 HV), and commercial fused silica glass (798.63 ± 76.76 HV).^[^ ^28^ ^]^ In this paper, we characterized the density of the sintered fused silica glass components in dependence of the printing strategy. Single‐wall structures reached full density (2.200 ± 0.005 g cm^−3^, 99.6 ± 0.5%). Fused silica parts printed with thicker walls built up from multiple strands did not reach full density (2 mm thick part, 2.133 ± 0.002 g cm^−3^, and 96.9 ± 0.2%), due to the presence of air inclusion between separate strands. Nevertheless, all fused silica parts showed isotropic shrinkage after sintering. The shrinkage of the FDM‐printed parts was characterized from printed green part to sintered fused silica glass. The shrinkage for a full density single‐wall component printed with a 40 vol% nanocomposite was measured to be 26.4 ± 0.2% in *x*–*y* direction, 25.7 ± 0.8% in the *z*‐direction and 25.5 ± 0.6% in wall thickness (three parts and three measurements each), which is in good accordance with the theoretical value of 26.3%. The small deviations in the measured shrinkage values are due to measurement inaccuracies resulting from the layered surface structure. Bulk components printed with the same 40 vol% nanocomposite showed a similar shrinkage of 26.6 ± 0.4% in the *x*–*y*‐direction and 26 ± 1% in the *z*‐direction (three parts and three measurements). This shows that, within the measurement's error margins, bulk components have the same shrinkage as full density single‐wall components and therefore allow a theoretical prediction of the shrinkage.

**Figure 5:**
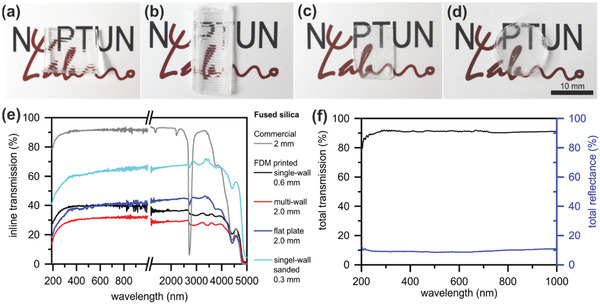
Optical characterization of FDM‐printed fused silica glass with different printing strategies. a) Optical transparency of a single‐wall‐printed fused silica glass (0.6 mm thickness). b) Optical transparency of a multiwall‐printed fused silica glass (2 mm thickness). c) Optical transparency of a single‐wall‐printed fused silica glass that was sanded in the green part stage (0.3 mm thickness). d) Optical transparency of a horizontally printed flat fused silica plate (2 mm thickness). e) UV–vis and FTIR spectra of FDM‐printed fused silica parts fabricated by different printing strategies compared to commercial fused silica glass. Transmission of FDM‐printed fused silica glass is lower than commercial fused silica due to increased scattering and reflection. f) UV–vis measurement of FDM‐printed single‐wall fused silica glass (0.6 mm thickness) using an integrating sphere setup to determine the effect of scattering and reflection in FDM‐printed fused silica. A high total transmission of up to 90% and a low reflection of <10% was measured, indicating a high amount of scattered light due to the layer structures.

The optical transparency of the 3D‐printed fused silica glass was determined using UV–vis and Fourier transform infrared (FTIR) spectroscopy (**Figure** **5**d). High surface roughness caused by the layered structure and the inclusion of small air cavities in objects with higher wall thicknesses built from multiple strands result in lower inline transmissions compared to commercial fused silica due to scattering and reflection of transmitting light. Highest transmission could be achieved for a layer height of 0.8 mm using the 0.8 mm nozzle with transmissions of >40% in the visible light range and up to 35% in the IR range for standing single‐wall structures (Figure 5a, 0.8 mm green part layer height and 0.6 mm wall thickness after sintering). Thicker multiwall parts (Figure 5b, 0.8 mm green part layer height and 2 mm thickness after sintering) decreased the transmission further. This is due to the fact that an increase in wall thickness is achieved by printing several strands besides each other, which increases the amount of FDM inherent defects such as air inclusions between strands as well as the amount of strand–strand boundaries. Higher wall thicknesses therefore show lower inline transmission due to increased light scattering. Flat structures printed horizontally on the print bed showed slightly higher transmissions of >40% at the same thickness (Figure 5c, 0.8 mm green part layer height and 2 mm thickness after sintering) since the bottom side, printed on the glass print bed, is substantially smoother due to a reduction of the layered structure. This can be used effectively to 3D‐printed flat objects with decent transparency, which can be used in applications such as, e.g., microfluidics. To further analyze the influence of scattering and reflection on the light transmission in FDM‐printed fused silica, we measured the standing single‐wall structure with an integrating sphere setup to determine total transmission as well as total reflectance, including both inline and diffuse transmission and reflectance (Figure 5e). We measured a high diffuse transmission of up to 90% and a reflectance of about 10% over a range from 300 to 1000 nm, therefore, indicating that the highest transmission losses can be attributed to scattering of the light on the layered surface.

To optimize the optical transparency we developed a manual postprocessing protocol for the printed green part. After printing, the layered surfaces were smoothened by sanding the printed part with sand papers (grit 1200 and 7000). Since the green parts behave like a polymer and are much softer and easier to process than glasses, the parts can be sanded and smoothed much easier than compared to conventional glass grinding and smoothing processes.^[^ ^10^ ^]^ After debinding and sintering, the sanded parts result in fused silica glass without the typical FDM layer structure having an improved optical clarity (see Figure 5d). Transmissions higher than 60% in the visible and IR ranges could be achieved by sand paper grinding a single‐wall component. To remove the layer structure of single‐wall components, about 50% of the wall thickness had to be sanded. More complex components might be smoothened using sand blasting techniques as has been shown before for other FDM‐printed materials.^[^ ^35^ ^]^

## 5 Conclusion

In this paper, we presented melt‐extrusion‐based AM shaping of transparent fused silica glass by FDM and FFD using thermoplastic silica nanocomposites, which can be subsequently converted to transparent fused silica glass in a heat treatment. In contrast to already established glass 3D printing mechanisms, FDM and FFD offer several advantages making previously inaccessible structures possible. The rapid solidification of the extruded molten feedstock allows printing of high‐aspect‐ratio structures without warping, deformation by viscous flow, or risk of cracking, as we could show by printing high and thin‐walled single‐wall container glasses with high aspect ratios of >480 for various potential applications such as laboratory equipment, lighting application, or components for everyday usage. FDM further offers the possibility of one‐step manufacturing of microfluidic chips without the need for additional bonding steps. Melt‐extrusion‐based AM, in general, is suited very well for multimaterial printing, as we could also demonstrate for our fused silica glass printing process by fabrication of multicolored glasses and simultaneous printing of polymeric support structures for complex overhanging features as well as print–pause–print modes allowing the integration of high‐resolution microstructures within the printed glass component. The printed glass showed a total transmission of >90% and an inline transmission of up to 40%. Smoothening the printed green parts using sandpaper allowed us to increase inline transmission of the final glass component up to >60%. Melt‐extrusion‐based AM represents a major addition to the field of fused silica glass 3D printing enabling novel applications in a great variety of fields from design, lighting, and jewelry to customized laboratory containers and microfluidic reactors.

## 6 Experimental Section

### Materials

PVB was purchased from Kuraray Europe GmbH. PEG was purchased from Merck. The metal salts for doping of fused silica glass (HAuCl_4_·*x*H_2_O (≈50% Au basis), CoSO_4_·7H_2_O, and Ce(NO_3_)_3_·6H_2_O were purchased from Merck. PLA filament for printing of support structures was purchased from Ultimaker, Netherlands.

### Nanocomposite Preparation

Thermoplastic fused silica nanocomposites with solid loadings of 40 and 50 vol% were produced according to a procedure described in the previous publication.^[^ ^26^ ^]^ For this, silica nanopowders having a mean diameter of 100 nm were mixed to a solution of PEG with a molar mass of 4000 g mol^−1^ and PVB in water using a laboratory dissolver of type RZR 2101 (Heidolph Instruments, Germany) equipped with a dissolver stirrer of type R 1302 (IKA, Germany). After mixing, the nanocomposite was dried at 65 °C for at least 2 days.

For printing of binary fused silica glasses, the nanocomposites were doped with metal salts (HAuCl_4_, Co(SO_4_), Ce(NO_3_)_3_, and 0.05 wt% in regard to silica) during the initial premixing step.

### Filament Preparation

The fused silica nanocomposite granules were plasticized in a twin screw extruder of type Teach‐Line ZK 25T (Collin, Germany) at 130 °C using a dosing unit of type K‐Tron K‐SFS‐24 (Cooperion, Switzerland) at a dosing speed of up to 1.5 kg h^−1^. The molten feedstock was extruded using a 2.85 mm die, cooled down in an air stream, and spooled continuously. The diameter of the obtained filament was measured ten times over a length of 10 m using a caliper to determine the mean diameter and its variance.

### Fused Deposition Modeling

FDM printing of the fused silica nanocomposite filament with a mean diameter of 2.85 mm was done using a modified commercial FDM printer (Ultimaker 3, Ultimaker B.V., Netherlands). The feeding motor was fixed directly on the print head instead of the back of the printer, as supplied, changing the filament feeding mode from bowden type to direct drive (see Figure 1c). The CAD models were sliced using the software Ultimaker Cura 4.8.0 to generate the G‐code file required by the FDM printer. Round nozzles of 0.4, 0.6, and 0.8 mm were used in the FDM printing process. Increasing the nozzle size reduced the printing duration but also decreased the resolution. Nozzles made of brass and hardened steel were used with no significant difference to be found. The general FDM printing parameters shown in **Table** **1** were used for all prints with some adaptations for specific prints as stated in the following.

**Table 1:** The general parameters for FDM printing of thermoplastic silica nanocomposites using various nozzle sizes

Parameters	0.4 mm nozzle	0.6 mm nozzle	0.8 mm nozzle
Nozzle temperature [°C]	160–180	160–180	170–190
Build plate temperature [°C]	60	60	60
Cooling fan speed [%]	100	100	100
Layer thickness [mm]	0.2–0.3	0.27–0.4	0.27–0.53
Line width [mm]	0.35	0.6	0.8
Infill [%]	0 or 100	0 or 100	0 or 100
Infill pattern	Lines	Lines	Lines
Material flow rate			
Wall [%]	100	100	100
Infill [%]	120	120	120
Top/bottom layer [%]	120	120	120
Printing speed			
Wall [mm s^−1^]	15–30	15–30	15–30
Infill [mm s^−1^]	30–60	30–60	30–60
Top/bottom layer [mm s^−1^]	30–60	30–60	30–60
Infill‐wall overlap [%]	10	10	10

The infill, which is the percentage of volume coverage within enclosed sections of the model, was set to either 100% for dense objects or 0% for hollow prints, depending on size, thickness, and application. High‐aspect‐ratio single‐wall structures were printed using the spiralize function of the slicer with 0% infill. For FDM printing of microfluidic chips, the parameters were slightly adjusted, by turning off the cooling fan expect for bridging layers and choosing a 100% infill. To print the bridging layer closing the microfluidic channel structure the flow rate was decreased to 50%, and printing speed was decreased to 12.5 mm s^−1^, and the cooling fan was set to 100% to minimize sagging.

Multimaterial printing was done by using the dual extrusion function of the FDM printer. To show the feasibility of multimaterial printing, a component consisting of fused silica nanocomposite with a small area in the middle was printed with metal‐salt‐doped nanocomposite filaments. The metal salt doping had no influence on the printing parameters. In addition, overhanging structures were stabilized by support structures printed with PLA using dual extrusion. The PLA support structures either fell off on their own, after aqueous debinding or could be removed by thermal decomposition prior to sintering.

The print–pause–print principle was shown by printing a customized bracket, with 100% infill, around an MLA made from the same silica nanocomposite. The MLA was fabricated by hot embossing of a thermoplastic nanocomposite substrate.^[^ ^34^ ^]^ For this, the nanocomposite substrate and a metallic hot embossing mold displaying the MLA structure were heated to 130 °C. The hot embossing mold was pressed onto the nanocomposite substrate with a pressure of about 4 N cm^−2^ for a duration of 10 s. After cooling down, the mold was removed to obtain the microstructured nanocomposite. This MLA was integrated into a 3D‐printed bracket using the print–pause–print principle. For this, a bracket having a cavity with the same size as the MLA was printed. The print was shortly paused after finishing the walls of the cavity, and the lens array was manually inserted. Afterward, the print was resumed to fully integrate the hot embossed microstructure component into the FDM‐printed part.

Smoothening of the surfaces to optimize optical clarity was shown by postprocessing of FDM‐printed single‐wall green parts: an FDM‐printed nanocomposite plate (single‐wall printing, 0.8 mm wall thickness) was sanded first with a sand paper (grit 1200) until the layer structure was removed. Afterward, the parts were sanded further with a finer sand paper (grit 7000) and then cleaned with deionized (DI) water.

### Fused Feedstock Deposition

The fused silica nanocomposite granules having a solid loading of 50 vol% were printed using a commercial FFD‐type printer FFD150H (3D Figo GmbH, Germany). The CAD models were sliced using the software Ultimaker Cura 4.8.0 to generate the G‐code file required by the FFD printer. A round brass nozzle with a diameter of 0.5 mm was used for all prints. The extruder and nozzle temperatures were set to 105 and 95 °C, respectively. The print bed was kept at room temperature. All parts were printed with a printing speed of 70 mm s^−1^ and a layer thickness of 0.3 mm.

### Debinding and Sintering

Debinding of the samples was done using a two‐step debinding protocol. The parts were immersed in water at 40 °C for a minimum of 5 h and dried at 65 °C for a minimum of 3 h afterward. In the second step, the remaining binder was removed by thermal decomposition in an ashing furnace of type AAF (Carbolite/Gero, Germany). The thermal debinding was done with a heating rate of 1 K min^−1^ and dwelling phases at the critical decomposition temperatures (270, 400, and 600 °C for 1 h each) in air. The debinded samples were sintered to dense and transparent fused silica glass using a high‐temperature tube furnace of type STF16/450 (Carbolite/Gero, Germany) for sintering in vacuum or a bottom loader furnace of type BLF 18/3 (Carbolite/Gero, Germany) for sintering under atmospheric pressure. The parts were sintered either in vacuum (1 × 10^‐1^ mbar) with a dwelling phase at 1320 °C for 2 h or under atmospheric air pressure with dwelling phases at 1250 and 1320 °C for 2 h each. Heating and cooling rates of 3 K min^−1^ were used, respectively, for both sintering protocols.

### Mechanical Characterization

Tensile tests were performed using a Zwick model Z005 (Zwick Roell, Germany) according to DIN EN ISO 527‐2 with a load speed of 1 mm s^−1^ at a temperature of 20 °C. To evaluate the influence of strand orientation, two different sets of samples were printed and evaluated with the strands being orientated either parallel or perpendicular to the strain direction. Injection‐molded samples were measured as a reference. The data were recorded and analyzed using the software “Zwick TestXpert II.”

Vickers hardness was measured using a microhardness tester of type Fischerscope HV 100 (Helmut Fischer GmbH, Germany) on sintered glass samples with a thickness of 1 mm. For the measurement, a load of 100 mN was applied for a duration of 20 s. Sintered fused silica glass with a thickness of 1 mm prepared by injection molding was used as reference.

Bending strength *f*_m_ was characterized using three‐point bending measurement on a Zwick model Z005 (Zwick Roell, Germany). FDM‐printed and FDM‐sintered glass bars with the dimensions of 40 × 5 × 1.7 mm were used for the measurements. Sintered fused silica glass samples prepared by injection molding having identical dimensions were used as a reference. The bars were placed on support rollers with a distance of 28.7 mm, and a load was applied in the middle using a load speed of 1 mm min^−1^ at a temperature of 20 °C until the sample ruptured. The data were recorded and analyzed using the software “Zwick TestXpert II.”

### Characterization of 3D‐Printed Fused Silica

The density *ρ* of the sintered glass parts was measured by the Archimedes principle using a lab scale Quintix 124‐1S and a density kit analytical balance YDK03 (Sartorius AG, Germany). The sintered fused silica parts were weighed in the dry state (*m*). Afterward, they were immersed in DI water (*T* = 20.5 °C) with a small amount of surfactant and the buoyancy mass *m*_b_ was determined. The density was calculated using following equation, with *ρ*_H2O_ being the density of water

(1) $$\rho \;=\frac{m\;\rho_{H_{2}O}}{-m_{b}}\;$$

The shrinkage was determined by measuring three different FDM‐printed parts in the green part stage and after sintering with a caliper. The theoretical linear shrinkage *Y*_s_ can be calculated in dependence of the solid loading *Φ*, theoretical density *ρ*_t_, and final density *ρ*_f_ of the manufactured object using following equation

(2) $$Y_{s}=1-{\left(\frac{\Phi }{\rho_{t}/\rho_{f}}\right)}^{\frac{1}{3}}$$

Optical inline transmission was determined by using a UV–vis spectrometer of type Evolution 201 (Thermo Scientific, Germany) and an FTIR spectrometer of type Frontier 100 MIR‐FTIR (Perkin Elmer, Germany). Total UV–vis transmission and reflectance were measured using a UV–vis–NIR spectrophotometer of type UV‐3600i Plus (Shimadzu, Japan) equipped with an integrating sphere attachment of type ISR‐1503 (Shimadzu, Japan). Fused silica glass slides (2 mm thickness, Toppan Photomasks, Inc., USA) were used as a reference sample for all measurements.

### Statistical Analysis

All data were given as the mean values ± standard deviation calculated from multiple measurements. All transmission spectra were shown with subtracted background and without any further corrections. Three‐point bending and tensile testing data were recorded and analyzed by TestXpert II. Other data were processed by either “Origin” or “Microsoft Excel.”

## Conflict of Interest

The Glassomer GmbH has patented the technology described within this paper (application/patent no. EP20195971.5) and is in the process of commercializing it. The authors declare no other competing interests.

## Supporting information

Supporting Information

## Data Availability

The data that support the findings of this study are available from the corresponding author upon reasonable request.
