# Supplementary material for: Melt‐Extrusion‐Based Additive Manufacturing of Transparent Fused Silica Glass
Source: Adv Sci (Weinh). 2021 Oct 20;8(23):2103180. doi: 10.1002/advs.202103180 (PMC8655167; doi:10.1002/advs.202103180)
Supplement: Supplementary file 1 — Supporting Information [file ADVS-8-2103180-s001.pdf]

WILEY-VCH

© 2021 Wiley-VCH GmbH

ADVANCED SCIENCE
Open Access

Supporting Information

for *Adv. Sci.,* DOI: 10.1002/advs.202103180

Melt extrusion-based additive manufacturing of transparent fused silica glass

Markus Mader, Leonhard Hambitzer, Phillip Schlautmann, Sophie Jenne, Christian Greiner, Florian Hirth, Dorothea Helmer, Frederik Kotz-Helmer, Bastian E. Rapp*

Supporting Information

Melt extrusion-based additive manufacturing of transparent fused silica glass

Markus Mader, Leonhard Hambitzer, Phillip Schlautmann, Sophie Jenne, Christian Greiner, Florian Hirth, Dorothea Helmer, Frederik Kotz-Helmer, Bastian E. Rapp*

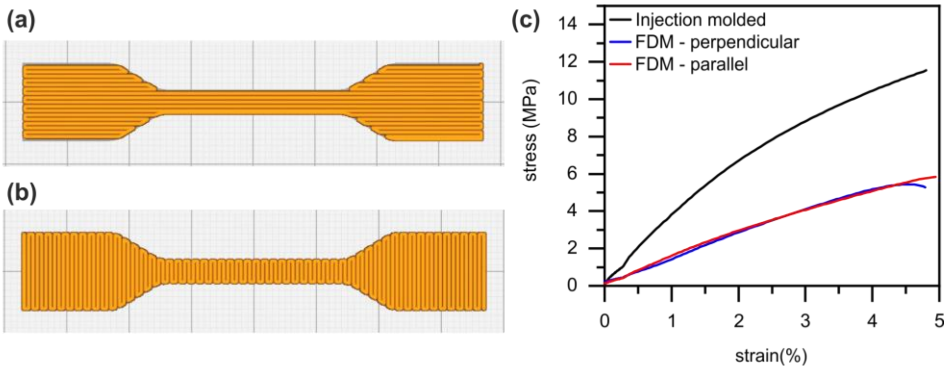

Figure S1. Mechanical characterization of nanocomposite green parts by tensile testing. **(a)** Schematic view of FDM printed tensile test specimen with strand orientation parallel to strain direction. **(b)** Schematic view of FDM printed tensile test specimen with strand orientation perpendicular to strain direction. **(c)** Exemplary stress strain curves of FDM printed nanocomposites with strands parallel and perpendicular to the strain direction in comparison to injection molded specimens.

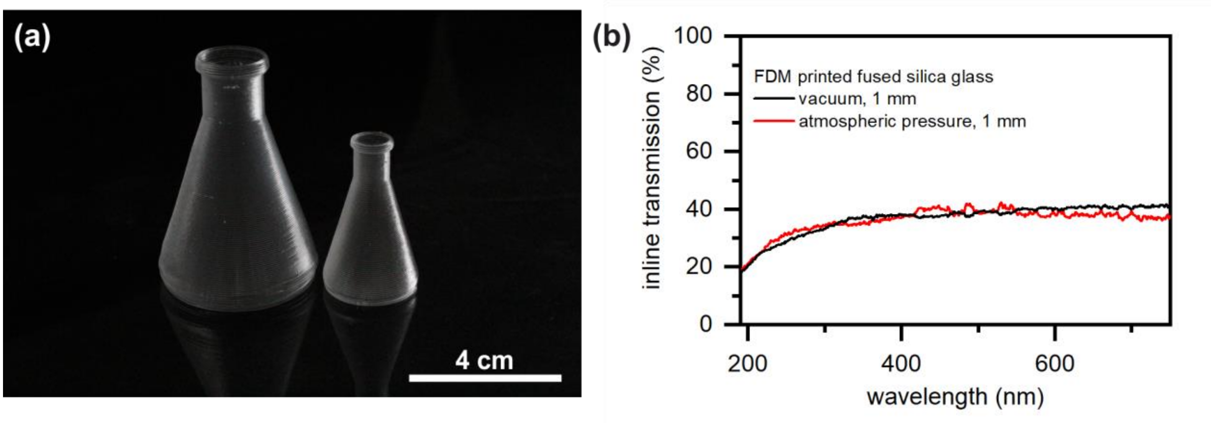

Figure S2. Comparison of vacuum and atmospheric pressure sintering. (a) The big flask was sintered under atmospheric pressure while the smaller flask was sintered under vacuum showing that the visual appearance is identical. (b) UV/Vis measurements of 1 mm thick samples sintered under atmospheric pressure and sintered under vacuum.

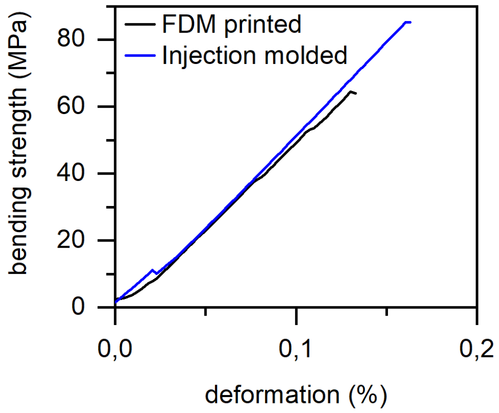

Figure S3. Exemplary 3 point bending measurements of injection molded and FDM printed fused silica glass in comparison.

Table S1. Tensile testing of FDM printed nanocomposite green parts to determine tensile modulus E_t elongation at breakage ε_b and breaking stress σ_b. The specimens were printed with all strands being parallel to the strain direction.

No.	E_t /MPa	ε_b /%	σ_b /MPa
1	101.985	3.583	5.840
2	125.123	3.529	5.435
3	140.965	2.961	5.538
4	148.055	3.435	5.479
5	135.956	3.382	6.164
6	139.317	2.536	5.006
Mean	132 ± 16	3.2 ± 0.4	5.6 ± 0.4

Table S2. Tensile testing of FDM printed nanocomposite green parts to determine tensile modulus E_t elongation at breakage ε_b and breaking stress σ_b. The specimens were printed with all strands being perpendicular to the strain direction.

No.	E_t /MPa	ε_b /%	σ_b /MPa
1	151.893	3.234	5.248
2	82.6596	3.509	5.439
3	80.4320	2.927	5.011
4	89.2752	3.067	5.585
5	139.423	3.344	5.283
Mean	110 ± 30	3.2 ± 0.2	5.3 ± 0.2

Table S3. Tensile testing of injection molded nanocomposite green parts to determine tensile modulus E_t elongation at breakage ε_b and breaking stress σ_b as a comparison to FDM printed parts.

No.	E_t /MPa	ε_b /%	σ_b /MPa
1	304.357	3.537	11.467
2	324.565	3.494	11.739
3	331.189	3.570	11.894
4	303.791	3.470	11.538
Mean	316 ± 14	3,52 ± 0.05	11.89 ± 0.19

Table S4. 3 point bending test of injection molded fused silica glass to determine the bending strength f_m of sintered glass.

No.	f_m /MPa
1	106.461
2	74.333
3	62.716
4	85.148
5	112.539
6	102.830
7	83.543
Mean	90 ± 18

Table S5. 3 point bending test of FDM printed fused silica glass to determine the bending strength f_m of sintered glass.

No.	f_m /MPa
1	58.450
2	81.873
3	62.905
4	87.164
5	52.818
6	77.232
7	62.348
8	55.417
9	64.391
10	70.610
Mean	67 ± 11

Table S6. Vickers hardness HV measurements of FDM printed fused silica glass in comparison to injection molded (IM) fused silica glass.

No.	HV - FDM	HV - IM
1	656.3	641.2
2	796.4	1089.7
3	727.1	802.2
4	643.6	669.3
5	568.8	679.1
6	997.0	668.3
7	1074.3	797.6
Mean	780 ± 190	760 ± 160
